# HCV Induces Telomerase Reverse Transcriptase, Increases Its Catalytic Activity, and Promotes Caspase Degradation in Infected Human Hepatocytes

**DOI:** 10.1371/journal.pone.0166853

**Published:** 2017-01-05

**Authors:** Zhaowen Zhu, Huy Tran, M. Meleah Mathahs, Thomas O. Moninger, Warren N. Schmidt

**Affiliations:** 1 Department of Internal Medicine and Research Service, Veterans Affairs Medical Center, Iowa City, IA, United States of America; 2 Department of Internal Medicine Roy G. and Lucille A. Carver College of Medicine, University of Iowa Iowa City, IA, United States of America; 3 Central Microscopy Research Facility Roy G. and Lucille A. Carver College of Medicine, University of Iowa Iowa City, IA, United States of America; University of Washington, UNITED STATES

## Abstract

**Introduction:**

Telomerase repairs the telomeric ends of chromosomes and is active in nearly all malignant cells. Hepatitis C virus (HCV) is known to be oncogenic and potential interactions with the telomerase system require further study. We determined the effects of HCV infection on human telomerase reverse transcriptase (TERT) expression and enzyme activity in primary human hepatocytes and continuous cell lines.

**Results:**

Primary human hepatocytes and Huh-7.5 hepatoma cells showed early de novo TERT protein expression 2–4 days after infection and these events coincided with increased TERT promoter activation, TERT mRNA, and telomerase activity. Immunoprecipitation studies demonstrated that NS3-4A protease-helicase, in contrast to core or NS5A, specifically bound to the C-terminal region of TERT through interactions between helicase domain 2 and protease sequences. Increased telomerase activity was noted when NS3-4A was transfected into cells, when added to reconstituted mixtures of TERT and telomerase RNA, and when incubated with high molecular weight telomerase ‘holoenzyme’ complexes. The NS3-4A catalytic effect on telomerase was inhibited with primuline or danoprevir, agents that are known to inhibit NS3 helicase and protease activities respectively. In HCV infected cells, NS3-4A could be specifically recovered with telomerase holoenzyme complexes in contrast to NS5A or core protein. HCV infection also activated the effector caspase 7 which is known to target TERT. Activation coincided with the appearance of lower molecular weight carboxy-terminal fragment(s) of TERT, chiefly sized at 45 kD, which could be inhibited with pancaspase or caspase 7 inhibitors.

**Conclusions:**

HCV infection induces TERT expression and stimulates telomerase activity in addition to triggering Caspase activity that leads to increased TERT degradation. These activities suggest multiple points whereby the virus can influence neoplasia. The NS3-4A protease-helicase can directly bind to TERT, increase telomerase activity, and thus potentially influence telomere repair and host cell neoplastic behavior.

## Introduction

Hepatitis C virus (*Hepacivirus*) (HCV) is a small, 9500 nucleotide, plus-stranded RNA virus that replicates with a single open-reading frame. The viral non-structural (NS) protein NS3-4A is a multi-functional protease-helicase and cleaves all viral NS proteins from the polyprotein downstream from the NS2-3 junction [1]. The protease domain comprises the amino-terminal third of NS3 and together with the NS4A peptide forms a classical serine-activated protease. In contrast, the carboxy-terminal 2/3 of the protein is a DExD-box “Super Family 2” (SF2) class RNA helicase that is essential for the viral life cycle. Mechanistically, the protease and helicase domains of NS3-4A are functionally interdependent for either enzyme to have optimal activity [2–4]. In general, RNA helicases are enzymes that unwind RNA using energy generated from a coupled ATPase and are required for many RNA reactions, such as mRNA processing, translation, and RNA protein binding. NS3 helicase is a member of the NS3/NPH-II group which includes enzymes that open RNA and in some cases DNA sequences and perform a variety of functions for host cells, viruses, and bacterial pathogens [5].

**Hepatocellular carcinoma** (HCC) caused by chronic HCV infection is a major cause of morbidity and mortality worldwide. The incidence of HCC in infected individuals is rising and the trend will likely continue because of aging and co-morbid risk factors [6]. While HCV infection is now almost fully treatable [7], only marginal progress has been made in understanding why the virus causes liver cancer. The oncogenicity of the virus must be better understood if we are to improve early detection and treatment strategies for HCC [8].

**Telomerase** is a specialized RNA-directed DNA polymerase that is activated in germinal, stem, and most malignant cells. The enzyme chiefly serves to lengthen and repair 3’ DNA telomeric strand overhanging ends that progressively shorten with each replication cycle because of the 3’ end replication problem [9]. Telomerase ensures that the telomeres at the chromosomal ends maintain the necessary length for additional replication cycles, thus protecting against chromosome inactivation from end to end fusion [10]. In rapidly dividing malignant cells, telomeres need constant repair so the enzyme, or an equivalent process, is reactivated to support high replication rates [11]. Because of the crucial role of telomerase for maintenance of chromosomal integrity there has been intense interest in numerous systems to determine why telomerase dysregulation occurs in cancer and other human diseases [12].

**Fig 1:**
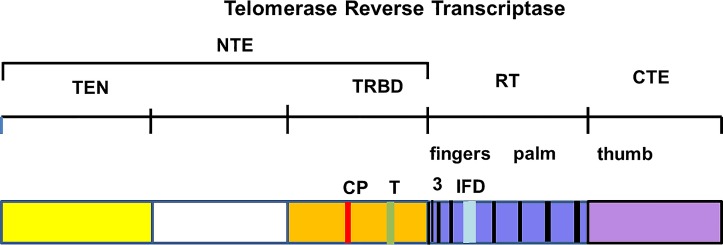
Structure of telomerase reverse transcriptase (TERT). TERT contains a long NTE^1^ linked with a central catalytic RT domain and a short carboxy CTE, (TEN, yellow; TRBD, orange; fingers, gray; RT, blue; CTE, magenta) with conserved motifs in TRBD (T, green; CP, red) or RT motifs (IFD, cyan) or black. The specific T and CP motifs in the TRBD recognize essential components of telomerase RNA. The specific motifs 3 and IFD are conserved areas located on surface of RT that likely interact with the active site through helical arrangements. ^*1*^*Abbreviations*: NTE = N-terminal extension; TEN = Telomerase essential N-terminal domain; TRBD = Telomerase RNA binding domain; RT = reverse transcriptase; CTE = C-terminal extension; IFD = insertion in fingers domain.

**Human** telomerase reverse transcriptase (TERT) is the core protein of a large ribonucleoprotein complex that contains the reverse transcriptase (RT) and a telomerase RNA component (TERC) which includes the telomeric template, structural elements, and TERT-binding RNA domains. The RT domain has classical polymerase motifs of fingers and palm regions; the latter including the active site with three catalytic aspartate residues (Fig 1). Three other domains are also present and include the telomerase essential N-terminal region (TEN), a telomerase RNA binding domain (TRBD) and a thumb/C-terminal extension (CTE) region [13, 14] (Fig 1). Modeling of TERT from other species has shown that the protein has a basic ring structure similar to other RTs with a palm active site, surrounded by sequences of CTE, TRBD, and RT surface motif 3 and insertion in finger domains (IFD). TERT provides the catalytic RT activity of the telomerase enzyme system which can be generated in vitro with incubations containing just the basic complex of TERT, TERC,and a telomeric DNA substrate. However, telomerase activity with telomere lengthening in the cell is generated within a high molecular weight, (> 19S, 1MD) ribonucleoprotein holoenzyme complex [15], which is tightly regulated in the nucleolus. The holoenzyme contains multiple regulatory proteins important for chromosomal recognition and telomere binding and enzyme performance [13] [16].

Our earlier work with telomerase showed that expression of TERT was increased in hepatocyte cultures after overexpression of HCV core protein as compared to normal human liver and uninfected cells. Increased expression of TERT was accompanied by increased TERT promoter activity, increased telomerase activity, and increased telomere length regulation in core-expressing cells [17]. Because HCV core protein is a known transcriptional activator of a number of host genes [18], it is most likely that core transcriptionally impacts telomerase expression leading to host cell responses that are vital for malignancy.

In the present work we hypothesized that study of the early events after HCV infection would confirm the interactions that we observed with core protein on the telomerase system, and identify additional potentially important events for hepatocarcinogenesis. We found that HCV infection of hepatocytes increases not only TERT expression, but directly influences telomerase catalytic activity, and triggers TERT degradation with caspase 7. These data further our understanding of the oncogenic capabilities of HCV and identify intriguing interactions of the virus with the telomerase system that may prove useful for the management and therapy of HCC.

## Methods and Materials

*Taq* DNA polymerase (*Perkin-Elmer Cetus*, Norwalk, CT), and Moloney murine leukemia virus reverse transcriptase (*Gibco/BRL Life Technologies*, Gaithersburg, MD) were used in these studies.

### Antibodies

Rabbit monoclonal antibodies to TERT having strict specificity for TERT C-terminal region were obtained from *EMD Millipore* (MABE14, clone Y182, 1:1000 dilution for WB analysis, 1:100 for immunofluorescence staining). Mouse monoclonal antibodies to NS3 and NS5A were both from *EMD Millipore* (MAB8691 and MAB8694, 1:1000 dilution for Western blot analysis, 1:100 for immunofluorescence staining). All secondary antibodies were purchased from *Santa Cruz Biotechnology*. Mouse monoclonal antibody to HCV core protein was obtained from *Abcam* (C7-50, 1:1000 dilution for Western blot analysis). Mouse monoclonal anti-Flag antibody was purchased from *Sigma-Aldrich* (F3165, 1:10,000 dilution for Western blot analysis). The loading control and fraction distinctive antibodies were as follows: anti-Actin antibody (*Sigma-Aldrich*, A2066, rabbit polyclononal, 1:1000 dilution for Western blot anaysis), and anti-GAPDH (*Abcam*, Ab8245, mouse monoclonal, 1:2000 dilution for Western blot analysis). All anti-caspase antibodies were obtained from *Cell Signaling*: Anti-caspase-6 antibody (#9762, polyclonal rabbit antibody, 1:1000 dilution), caspase-7 antibody (#9492, rabbit polyclonal, 1:1000 dilution), cleaved caspase-6 (Asp162) antibody (#9761, rabbit polyclonal, 1:1000 dilution), and cleaved caspase-7 (Asp198) (D6H1) antibody (#8438, rabbit monoclonal 1:1000 dilution). For immunfluorescence staining, goat anti-rabbit and mouse IgG (H+L) secondary antibodies, alexa fluor 488 and 568 conjugates, were obtained from *ThermoFisher Scientific* (A-11008, A-11001, A-11036 and A-11031, 1:1000 dilutions for all), and TO-PRO-3 iodide was also obtained from *ThermoFisher Scientific* (T-3605, 1:1000 dilution).

### Cell lines and cell culture

Primary human hepatocytes (PHH) were purchased from *Lonza Cell Culture Products*, *(*Basel Switzerland) and cultured following the manufacturer’s instructions. The infectious HCV cell culture (HCVcc) strain (J6JFH) as described [19] was inoculated into PHH or Huh-7.5 cells using published protocols [20]. The human hepatoma cell line (Huh 5–15) with replicating sub-genomic HCV RNA (genotype 1b) (Huh 5.15NS) [21] was cultivated as described [22]. Huh-7.5 cells harboring full length (Huh-7.5FL) Con1 (genotype 1b) replicons were passed as recommended by their laboratory of origin [23]. Wild type HEK-293 cells were purchased from University of Iowa Tissue Culture stores and passed routinely in minimal essential medium containing 10% fetal bovine serum.

### Vectors and constructs

pcDNA3.1 plasmid (*Invitrogen*) cloning strategies for HCV proteins have been described previously [17, 24]. Full length HCV NS3-4A genotype 1B sequences (nt 3420–5274) and wild type linked NS5A/5B sequences (nt 6258–9377) were amplified by RT-PCR from Con1B replicon RNA using appropriate flanking primers and cloned into expression vector. An enzymatically inactive mutant of NS3-4A was prepared by changing the catalytic serine of the NS3 catalytic site (serine #1165; HCV-1b) to an alanine with site directed PCR [24]

NS3-4A fragments consisting of protease, or helicase domains 1, 2 or 3 and NS4A were amplified by PCR from cloned genotype 1B sequences and inserted into a pcDNA 3.1 plasmid containing N-terminal FLAG sequences using appropriate restriction enzymes. All cloned fragments were verified by sequencing in the forward and reverse directions. The sizes and locations of the peptide fragments are depicted in the figures. A similar strategy was used to clone specific fragments of TERT with N-terminal FLAG sequences into p3XFLAG-CMV-10 vectors. Full length human TERT sequences and various sized fragments of TERT were generated from the catalytically active TERT plasmid pCI neo-hEST2. This plasmid and Telomerase RNA component (TERC) plasmid (pBS U3-hTR-500) were obtained from *Addgene (*MA).

### Isolation of RNA

Total RNA was extracted from cells using *Trizol* reagent (*Invitrogen)*, treated with Turbo RNase free DNase (*Ambion*, TX), and processed as described [22]. The cDNA was synthesized with superscript first-strand synthesis system (*Invitrogen)*. Real-time RT-PCR for HCV and TERT mRNA was performed using Taq DNA polymerase with the SYBR green Universal PCR Master Mix Protocol (*Perkin Elmer Applied Biosystems*, Foster City, CA). Quantitation was performed using the Comparative Cycle Threshold (ΔC_T_) method using GAPDH housekeeping gene as standard as described previously [25].

### Quantification of telomerase activity.

*Real Time* quantification of the basic Telomeric Repeat Amplification Protocol (TRAP) was performed essentially as we described [17]. Quantitative Telomerase detection kit (*US Biomax*, Inc) was used to measure telomerase reverse transcriptase enzymatic activity in cellular lysates according to manufacturer’s directions. As directed, “Relative Telomerase Activity” was calculated from a standard curve of reference samples and data were analyzed using relative fluorescence units as compared to controls. In some cases, telomerase reaction products were visualized using TRAPeze system, (*EMD Millipore*) 12% non-denaturing PAGE, and SYBR fluorescence labelling of products of the RT-PCR reaction.

### Western blot analyses

2x Laemmli electrophoresis sample buffer and supplies were from *Bio-Rad*, (CA). Western blots (WB) were performed as previously described using enhanced chemiluminescence for signal detection (*ECLTM*, Amersham) [26].

#### Immunofluorescence labelling

HCV replicons or infected Huh-7.5 cells were grown attached to coverslips, washed in PBS, fixed in absolute methanol, re-washed in PBS, then incubated with anti-TERT or anti-NS3-4A antibodies for 1 hr. Fluorescence labelling was conducted by one hour incubation of slides with secondary antibodies conjugated *to Alexa Fluor 488* (green) or *Alexa Fluor 568 (red)* fluorochromes. Slides were mounted with *Vectashield* (*Vector Labs*) containing TO-PRO-3 iodide to visualize nuclei *(ThermoFischer Scientific*). Confocal microscopy was performed on a *Zeiss LSM710* confocal fluorescence microscope. Mitochondrial labelling was performed using *MitoTracker* (*ThermoFisher*) Scientific.

### Immunoprecipitation

Immunoprecipitation was performed as described previously [20], with minor modifications. Briefly, log-phase HCV infected cells, Huh5-15NS replicons, or vector transfected HEK-293 cells were harvested, washed in PBS, lysed in cell lysis buffer (*Cell Signaling Technology*, Beverly, MA) and clarified by cold centrifugation (14,000xg for 10 min). An aliquot of supernatant containing about 500 μg protein was incubated with 2μg anti-NS3 monoclonal antibody (*Meridian Life Science*, Saco, ME) or anti-hTERT antibody MABE14 (*EMD Millipore*, MA) at 4°C overnight with gentle mixing. Then, 20 μl of recombinant Protein G Agarose (*Invitrogen*, CA) was added and incubated at 4°C for 3hrs. Immunoprecipitates were collected by centrifugation at 3,000 rpm for 30s at 4°C, washed three times with ice-cold PBS, then dissolved in 40μl 2x Laemmli electrophoresis sample buffer (*Bio-Rad*, CA) and assayed by WB. Normal rabbit or mouse IgG was always used as control (*Santa Cruz*, CA). In some cases, cell lysates were incubated with RNAases A or H (*Life Technologies*, NY); (50 or 100 μ/ml, 37°C, 20 min and 0.2 u/ml 37°C, 20 min, respectively) or DNAase 1, (*Qiagen*, CA) (70 μ/ml at room temperature, 1 hr), or ethidium bromide (0.1 mg/ml at room temperature, 1 hr) prior to immunoprecipitation.

### Luciferase TERT promoter studies

The Dual Reporter Gene System for assessment of TERT promoter activation was from *Promega*. A TERT promoter fragment spanning –255 to +40, designated pBT-255, was generously provided by Dr. Richard Hodes (National Institute on Aging). 100 ng/ml of pBT-255 or pGL3-basic vector were transfected with *Lipofectamine*^*T*M^ 2000 (*inVitrogen)* according to the manufacturer’s protocol. 1ng/ml of pRL-CMV coding for *Renilla* luciferase was co-transfected for calculation of transfection efficiency. 48 hr post transfection reporter lysis buffer was added and incubated at room temperature for 15 min before centrifugation to remove cell debris. For the assay, 20 μl of cell lysate was mixed with 100 μl of luciferase substrate and light emission was measured with the LumiCount Luminometer (*PerkinElmer Life Sciences*). Relative luciferase quantification was as described after correction for transfection efficiency with *Renilla* luciferase [17].

### 10–30% Glycerol gradient centrifugation

Isolation of TERT holoenzyme high molecular weight complexes was accomplished using 10–30% glycerol gradient centrifugation essentially as described [16]. Linear 10–30% glycerol gradients were formed in buffer G (25mM HEPES-KOH, 150mM KCl, 1.5mM MgCl2, 0.01% Tween-20, 10–30% glycerol, pH 7.5 supplemented with 5mM 2-mercaptoethanol, 10mM NaF, 1mM Na3VO4 and 1mM bezamidine) using a linear gradient gel mixer. Log-phase HCVcc infected Huh-7.5 or Huh5-15 NS replicon cells were lysed in ice-cold NP-40 buffer: 25mM HEPES-KOH, 150mM KCl, 1.5mM MgCl_2_, 10% glycerol, 0.5% NP-40, 5mM 2-mercaptoethanol, 0.1mM PMSF. Then, 500μl of supernatant aliquots (1 mg protein) were layered onto chilled 10 ml preformed glycerol gradients. Gradients were centrifuged at 35,000xg for 15hrs at 4°C using SW-41 rotor. Gradients were fractionated from the top removing sequential 450 ul aliquots. Thyroglobulin (670 kD, 19.3S) and aldolase (158 kDa, 7.3S) were separated on parallel gradients for size markers. 30μl of fractions were analyzed by WB or *Real Time* TRAP assays.

### Reconstituted cell free telomerase assays and NS3-4A

Reconstituted cell free assays tested the direct effects of NS3-4A on telomerase activity of both basic catalytic complexes of TERT and TERC, and high molecular weight holoenzyme complexes isolated on 10–30% glycerol gradients. For assay of the basic catalytic complex, TERT, TERC, and NS3-4A, were synthesized in separate tubes containing rabbit reticulocyte lysate (RRL), (*TNT quick coupled transcription/translation*, *Promega*) mixtures using designated expression vectors. TERC was generated from T7 runoff transcripts as described by the manufacturer, followed by RNA isolation and gel purification before addition to the TRAP assay. Optimal 2 ul aliquots of TERT and TERC (determined empirically) were assayed with varying amounts of NS3-4A by *Real time* TRAP or TRAPeze system to directly visualize telomeric sequences. For assay of holoenzyme complexes, 2 ul aliquots of high molecular weight gradient fractions were mixed with varying amounts of RRL synthesized NS3-4A in TRAP reactions and telomerase activity determined as described for the basic catalytic TERT complex.

### Statistics

Data from individual experiments as well as combined data from separate experiments were expressed as mean +/- standard error of the mean. The significance between means was determined using Student’s t-test with ANOVA using pooled variances. P values less than 0.05 were considered significant. All experimental findings, whether performed singly or in parts were repeated at least three times.

## Results

**Fig 2:**
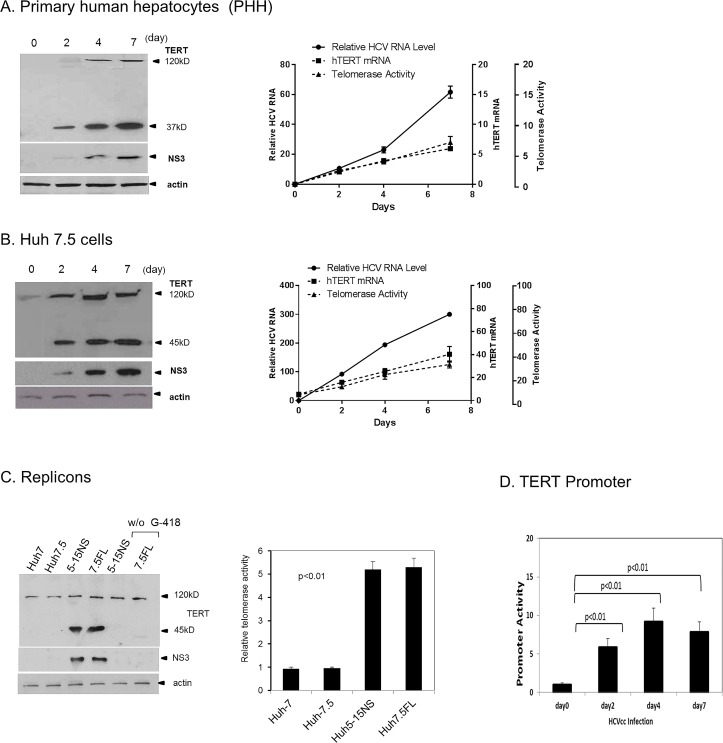
TERT activation and expression after HCVcc infection. **(A) Primary human hepatocytes (PHH) infected with J6JFH (HCVcc)** *Right panel*: HCV RNA and TERT mRNA were assayed in RNA isolates using quantitative RT-PCR. Telomerase activity was assayed in cellular lysates by *Real-time* Telomeric Repeat Amplification Protocol-Reverse Transcriptase PCR (TRAP-RT PCR) from cells collected at the indicated days post infection. HCV RNA p<0.05; day 2 vs 4 and p<0.01 day 7 vs 4. *Real-time* Telomerase enzyme activity p<0.01 all points as compared to day 2. TERT mRNA p<0.01 all points as compared to day 1. *Left panel*: WB analyses of cellular lysates for TERT protein and HCV NS3 taken on the indicated days post infection. **(B) Huh-7.5 cells infected with HCVcc**. *Right panel*: HCV RNA and TERT mRNA were assayed in RNA isolates using quantitative RT-PCR. Telomerase activity was assayed in cellular lysates by *Real-time* TRAP-RT PCR from cells collected at the indicated days post infection. HCV RNA p<0.01 all points as compared to day 2. TRAP activity p<0.01 all points as compared to day 0. TERT mRNA p<0.01 all points as compared to day 1. *Left panel*: WB analyses of cellular lysates for TERT protein and HCV NS3 taken on the indicated days post infection. **(C) WB and TRAP analysis in Non-structural (NS) or Full length (FL) HCV replicon lines.** *Left panel*: Log phase cultures were processed for WB analysis. Protein-blotted membranes were probed with anti-TERT (C-terminal specific) or anti-NS3 antibodies. Passage of replicons without G-418 selection medium for > 5 days (right two lanes) led to loss of HCV infection (absence of NS3-4A) and expression of 45 kD TERT fragment. *Right panel*: TRAP activity was assayed in replicon or control (Huh-7 or Huh-7.5) cell lysates by *Real-time* RT-PCR in 2–3 day log phase cultures. (TRAP activity of control cells < replicons p<0.01). **(D) HCVcc infection enhances TERT promoter function**. pBT255-luc (100ng/ml) and pRL-CMV (Renilla) (2ng/ml) plasmid were co-transfected into permissive Huh-7.5 cells. After 24 hrs 1.0 Multiplicity of Infection (MOI) of HCVcc was added to the cultures. The luciferase activity in cell lysates was determined at various times thereafter with dual luciferase reporter assay. p<0.01 all bars, as compared with day 0.

After infection of primary human hepatocytes (PHH) or permissive Huh-7.5 cells with cell culture strain of HCV (HCVcc) (Fig 2A and 2B respectively), there was increased TERT mRNA and protein expression as well as augmented TRAP activity that coincided with the rise of HCV RNA replication. As expected, replication in Huh-7.5 cells was more robust than PHH, but the time course of viral detection and appearance of TERT was quite similar in either cell type at 2–4 days. PHH did not express detectable TERT nor telomerase activity until after infection. As we reported previously [17], like all human hepatoma cell lines, Huh-7.5 cells produce some TERT regardless of infection, but the amount was increased over 100% (by density analysis, not shown) by 2 days after HCV infection. Both replicon lines (5-15NS and 7.5FL) showed modestly more TERT and significantly more telomerase activity than their uninfected parental cell lines (Fig 2C).

Human TERT exists as a native dimer in vivo and the full length monomer is known to size at about 120 kD on SDS gel electrophoresis [14]. After infection both PHH and Huh-7.5 cells showed the appearance of lower molecular weight species of immunoreactive TERT of 37 and 45 kD (Fig 2A and 2B respectively). The location of the fragments from the C-terminal end of TERT was deduced from WB probed with site-specific TERT antibody (C-terminal or N-terminal specific) and specific end-labelling experiments with FLAG or HA tags (S1 Fig). As shown later, the fragments likely result from caspase activation and targeting of TERT. The 45 kD species was lost in both replicon lines when passed in medium without G-418 selection (>5 days) concomitantly with the loss of HCV protein (NS3) expression (Fig 2C left panel).

Because transcriptional regulation of the TERT catalytic subunit is a major control mechanism for TERT expression [27] we also assessed TERT promoter activation following HCV infection in Huh-7.5 cells. TERT promoter was significantly activated early after infection and displayed a time course corresponding closely with the appearance of TERT mRNA and new protein (Fig 2D). These data also confirm our earlier findings which showed that HCV core protein elicited telomerase promoter activation in core-transfected cells [17].

**Fig 3:**
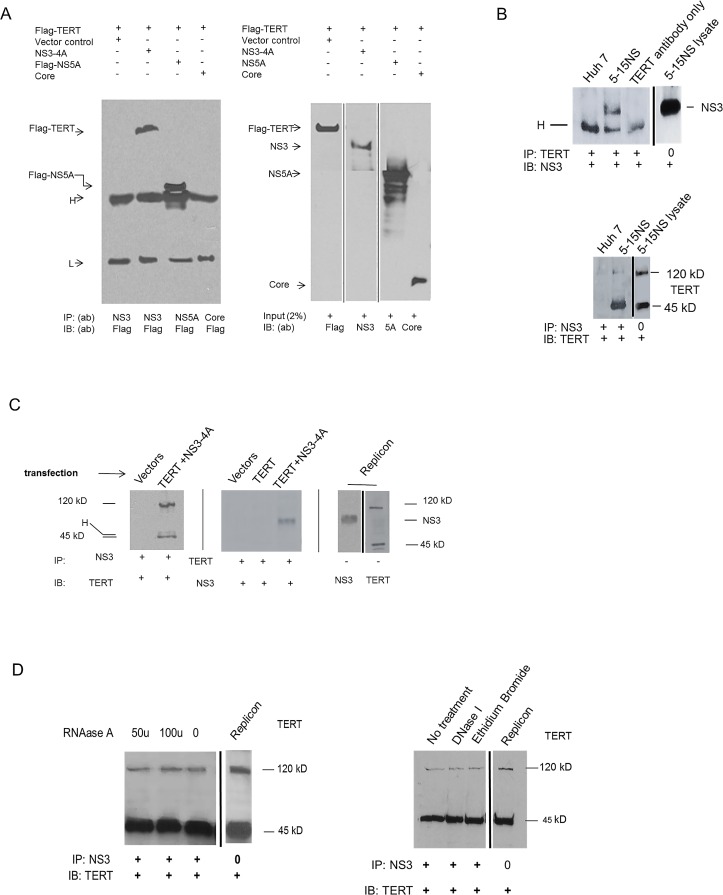
Immunoprecipitation: NS3-4A binds TERT. **(A)** N-FLAG-labelled TERT plasmid was transfected into HEK-293 cells together with vector only or vectors containing NS3-4A, N-FLAG NS5A, or core. FLAG-NS5A served as internal FLAG control to ensure TERT did not bind FLAG. Cellular lysates were then immunoprecipitated *(IP) with the indicated antibodies and the IPs evaluated on WB visualized with anti-FLAG antibody. Right panel shows sizing and verification of input protein on WB stained with indicated Immunoblot antibody (IB). **(B)** Log-phase replicons (Huh 5-15NS) or **(C)** vector transfected HEK-293 cells were harvested, lysed in cell lysis buffer, and then incubated with the indicated antibodies for immunoprecipitation. The IPs were subjected to gel SDS electrophoresis and the products assessed on WB using specific antibodies. **(D)** Cell lysates from Huh 5.15NS replicons were treated with RNAase A, DNAase I, or ethidium bromide prior to immunoprecipitation. The IPs were evaluated on WB stained with the indicated IB antibody. RNAase H (not shown) also had no effect. ^*^Abbreviations: (IP) = immunoprecipitation antibody. (IB) = Immunoblot antibody. Anti-TERT antibody = rabbit monoclonal specific to C-terminal end of TERT. Anti-NS3 antibody = rabbit polyclonal antibody. H = heavy Immunoglobulin chain, L = Light immunoglobulin chain. Immunoglobulin bands were expected in some cases from reactivity of second antibody for immunoglobulin in the IP.

Immunoprecipitation experiments were conducted to determine whether viral proteins specifically bind TERT. Using extracts from cells transfected with N-FLAG-TERT together with NS3-4A, core, NS5A or linked NS5A-NS5B (not shown) we found that only anti-NS3 antibody co-precipitated TERT (Fig 3A). Next, we confirmed that either anti-TERT (C-terminal specific) or anti-NS3 antibodies could precipitate both proteins from HCV infected cellular lysates (Fig 3B) or from HEK-293 cell lysates after transfection of either protein (Fig 3C). We also noted that anti-NS3 antibodies precipitated both 120 kD full length TERT as well as the 45 kD TERT fragment (Fig 3B and 3C). NS3-4A binding to TERT was resistant to DNAase and RNAases (A and H) treatments as well as the DNA intercalating agent ethidium bromide, suggesting that RNA or DNA sequences do not directly participate in the NS3-4A and TERT binding interaction (Fig 3D).

**Fig 4:**
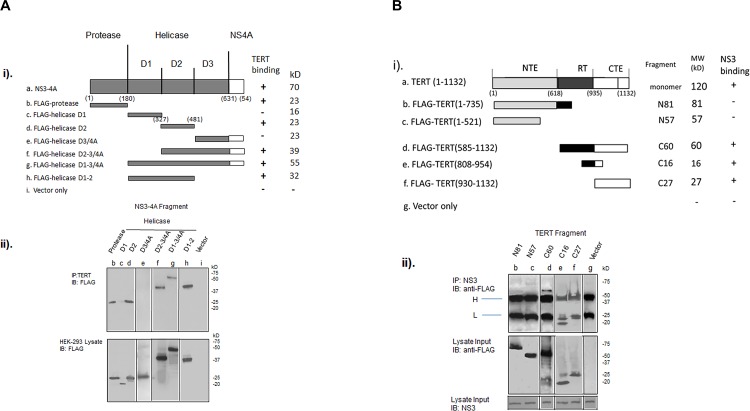
NS3-4A: TERT binding sites. **(A) Binding of NS3-4A fragments to endogenous TERT**. (i). Fragments of NS3-4A were constructed in 3xFLAG-CMV vectors with an N-terminal FLAG tag. ii). Following transfection of FLAG-NS3-4A fragments into HEK-293 cells, immunoprecipitation (IP) for endogenous TERT was performed using anti-TERT antibody and the complexes evaluated on WB using anti-FLAG antibodies (upper blot). Sizing of N-FLAG NS3-4A fragments after transfection on immunoblots stained with anti-FLAG antibody (lower blot). Abbreviations are as listed in Fig 3. **(B)** Binding of TERT fragments to NS3-4A. (i) Fragments of TERT were constructed in p3XFLAG-CMV-10 vectors with an N-terminal FLAG tag. (ii) The vectors were transfected into HEK-293 cells together with pcDNA3.1 NS3-4A and immunoprecipitation (IP) was performed using anti-NS3-4A antibody. IPs were evaluated on immunoblots using anti-FLAG antibodies (upper panel). Appropriate sizing of N-FLAG TERT fragments was on WB stained with anti-FLAG antibody (lower panel). Abbreviations are as listed in Fig 3.

To further define the binding interaction between NS3-4A and TERT, N-FLAG labelled fragments of NS3 and TERT were cloned, sequenced, and expressed in HEK-293 cells. Immunoprecipitation experiments were then used to map the approximate sites of binding between the two proteins (Fig 4A and 4B). Experiments with transfected N-FLAG NS3-4A fragments and immunoprecipitation of endogenous TERT from HEK-293 cells revealed that TERT bound NS3 at domain 2 helicase and/or protease sequences of NS3 (Fig 4A). Similarly, using N-FLAG TERT fragments, we noted that NS3-4A bound to the RT/CTE region of TERT and not to the N-terminal extension (NTE) regions (Fig 4B).

**Fig 5:**
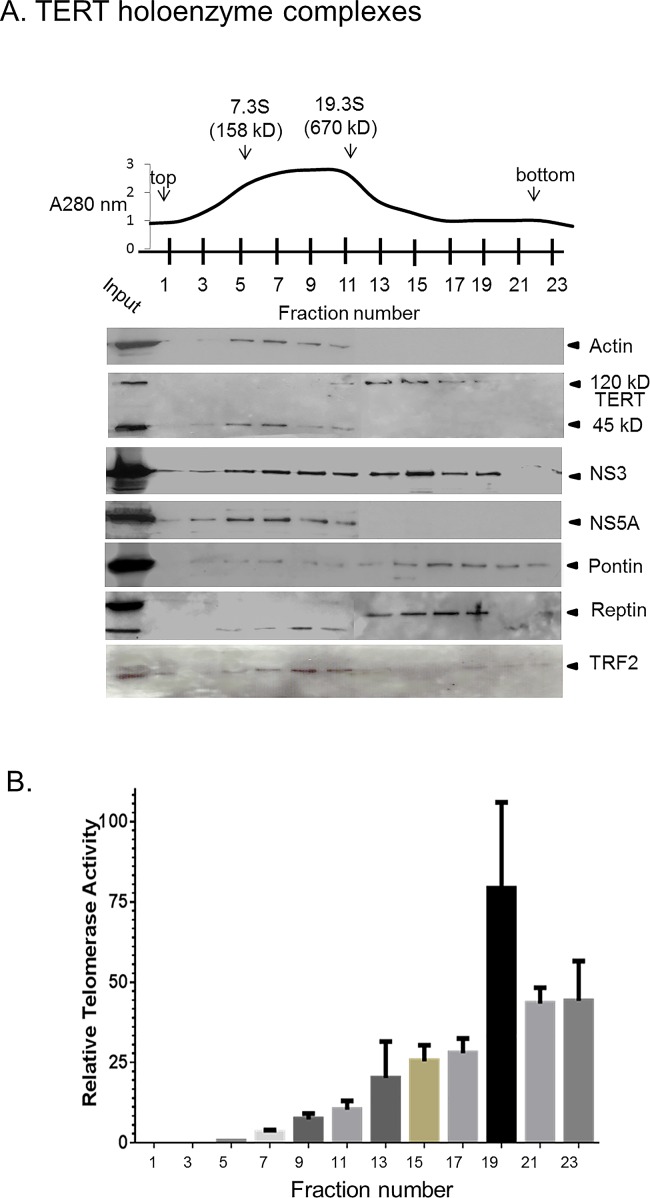
Binding of NS3-4A to TERT holoenzyme complexes. **(A) NS3-4A association with TERT holoenzyme complexes**. *NP-40 buffer* extracts from Huh-5.15NS replicons were subjected to 10–30% glycerol gradient centrifugation as described in Methods. Gradients were then fractionated into 400ul fractions and 10ul aliquots of each were analyzed on WB using specific antibodies to the indicated proteins. **(B) Telomerase activity in holoenzyme gradient fractions**. 10 μl aliquots of each gradient fraction (A) were subjected to TRAP-RT-PCR assay in triplicate and quantified relative to input signal telomerase activity. Each bar represents the mean +/- SEM. Relative telomerase activity of fractions > 11 vs fractions < 11, p < 0.001.

Glycerol gradient sedimentation [15, 16] was used to determine whether NS3-4A was bound to catalytically active, heavy (> 1 MD) telomerase holoenzyme nucleoprotein complexes in HCV infected cells (Fig 5A). WB analysis of gradient fractions showed that only full length TERT was recovered with holoenzyme complexes (Fig 5A) and these fractions retained maximal enzymatic TRAP activity (Fig 5B). In contrast, the 45 kD TERT fragment was recovered entirely with lower molecular weight material that contained much less TRAP activity. This finding suggests that the fragment is not a component of the holoenzyme complex and consequently it is unlikely to be a source of canonical telomerase activity in the cell. NS3-4A was detected in both high and low molecular weight fractions consistent with its ability to bind full length 120 kD TERT as well as its primary role in cytoplasmic viral replication (Fig 5A). In contrast, NS5A, which showed no TERT binding ability was restricted to just low molecular weight fractions. The integrity of the holoenzyme complexes that we isolated was further verified with detection of the ATPases Pontin and Reptin in the high molecular weight fractions which is consistent with their role as holoenzyme components [16]. Additionally, TRF2, a shelterin complex subunit which does not bind TERT [28] was mainly found in low molecular weight fractions although it was faintly detected in very heavy fractions consistent with its ability to form RNA and DNA-protein complexes [29, 30](Fig 5A).

**Fig 6:**
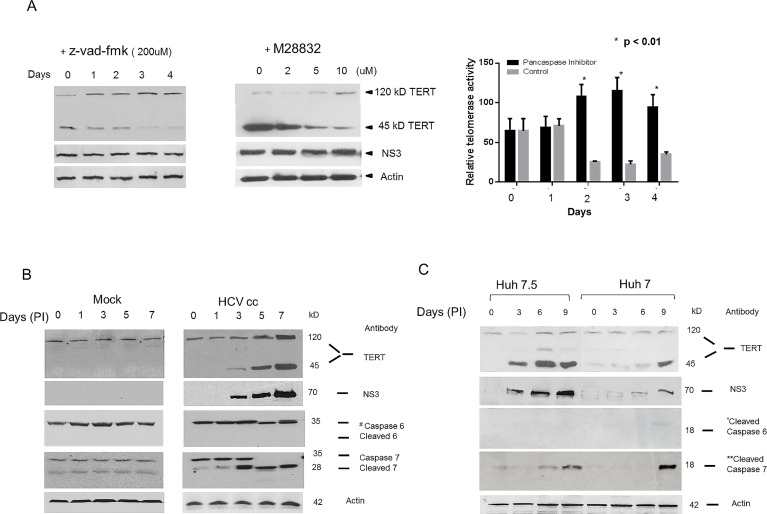
HCV infection triggers Caspase degradation of TERT. **(A)** Left panel, log phase Huh 5.15NS replicons were incubated with the pancaspase inhibitor (z-vad-fmk) (200uM) and at various times cellular lysates were assayed on WB for TERT or NS3-4A using C-terminal specific antibody. In right panel, log phase Huh 5.15NS replicons were incubated with indicated amounts of the specific Caspase 7 inhibitor (Millipore 28832) for 48 hr. Cellular lysates were then assayed on WB for TERT using C-terminal specific antibody. In the lower panel, log phase Huh 5.15NS replicons were incubated with pancaspase inhibitor or vehicle control for the indicated times and relative telomerase activity was determined in the cellular lysates as compared to day 0 using *Real-time* TRAP-RT PCR. *[Pancaspase inhibitor > control days 2, 3, and 4, p< 0.01] **(B)** Huh-7.5 cells were either infected with HCVcc (right panel) or mock control (left panel) and on various days WB were performed to detect TERT, NS3, and un-activated full length caspases 6 and 7 as well as cleaved fragments as indicated. ^#^Antibodies recognizing both full length and upper cleaved caspase fragments were from *Cell Signaling* (Caspase 7 #9492 and Caspase 6 #9762). **(C)** Huh-7.5 or Huh-7 cells were infected with HCVcc and on various days assayed for TERT as well as lower kD cleaved fragments of caspase 6 and 7 by WB. *Cell Signaling* *ASP 162 antibody (#9761) was used to detect cleaved caspase 6 and **ASP 198 antibody (#8438) was used to detect cleaved caspase 7 with products appearing at 18 kD in both cases.

In the next experiments we investigated whether the 45 kD fragment arises, in part, by caspase activation known to occur after HCV infection [31, 32] (Fig 6A). TERT is also a known target for effector caspases 6 and 7 [33]. WB experiments showed that the amount of 45 kD fragment was markedly reduced while the 120 kD monomer was bolstered in replicon cultures incubated with either the pancaspase inhibitor, Z-vad-fmk, or the specific caspase 7 inhibitor, *Millipore* 28832, (Fig 6A, left and right panels respectively). Furthermore, pancaspase inhibitor added to replicon cells maintained and even increased telomerase activity in maturing cultures of Huh 5.15NS replicons (Fig 6A, lower graph) linking increased telomerase activity with increased undegraded TERT monomer. Activated caspase fragments of 7 but not 6 appeared early after HCVcc infection of the highly permissive Huh-7.5 cells (Fig 6B and 6C) and much later in the less permissive Huh-7 wild type cells. Nonetheless, in either line the appearance of the 45 kD TERT species was concomitant with appearance of HCV proteins. It was interesting that caspase 6 was not activated after infection, in spite of the fact that TERT is a known target for caspase 6, [33], thus suggesting specificity for the HCV activation of caspases to target TERT.

**Fig 7:**
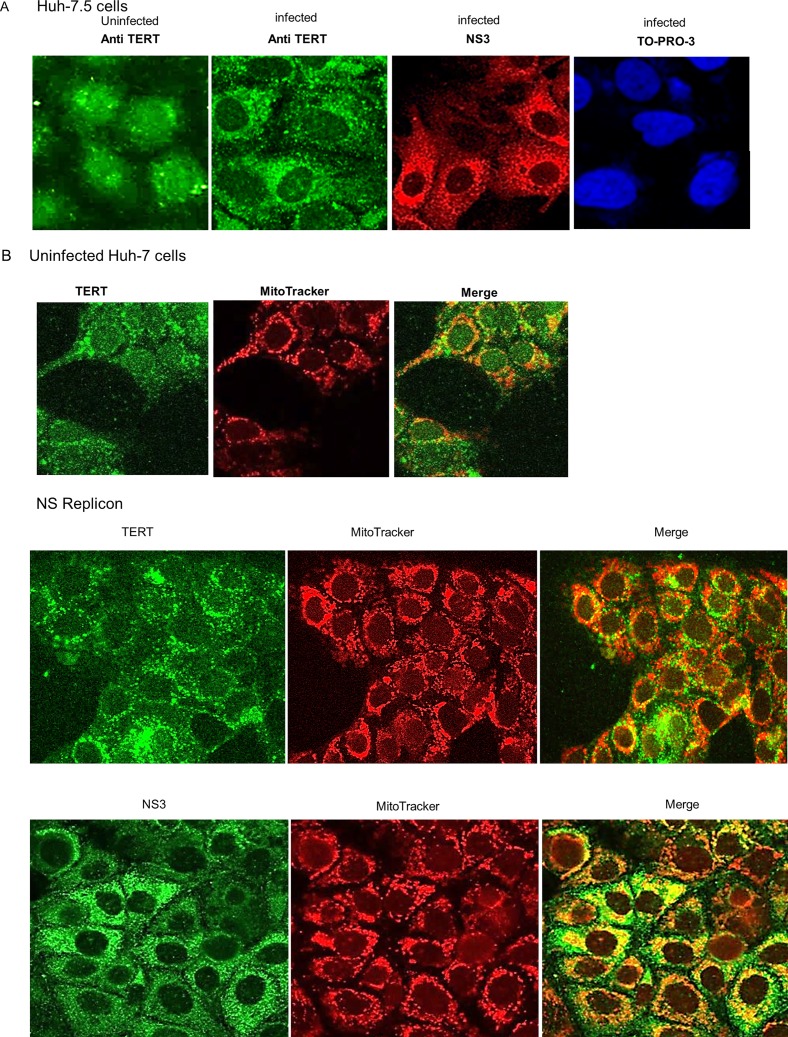
Immunocytochemical localization of TERT and NS3. **(A)** Uninfected or HCV infected log-phase Huh-7.5 cells were reacted with anti-TERT or anti-NS3 antibodies, then with Alexa Fluor 488 (green) or Alexa Fluor 568 (red) labelled second antibodies respectively. TO-PRO was used to visualize nuclei (lower panel). **(B)** Uninfected log phase Huh 7 cells (upper panel), were labelled with anti-TERT then co-labelled with *MitoTracker* (red). Confocal microscopy to merge images was performed on a *Zeiss LSM710* confocal fluorescence microscope. Merged fluorescence is yellow. Log-phase Huh-5.15 replicons were labelled with anti-TERT (middle panel) or anti-NS3 (lower panel) antibodies, (green), then co-labelled with *MitoTracker*. Confocal images were generated as above.

Immunofluorescence labelling for TERT after HCV infection was performed on fixed cells in situ (Fig 7A and 7B). In uninfected Huh-7.5 cells, TERT showed perinuclear cytoplasmic foci and diffuse nuclear staining (Fig 7A). After viral infection, TERT staining increased and localized prominently to perinuclear granules. Most cellular staining for NS3 was a strong cytoplasmic pattern with perhaps wispy nuclear staining.

Fragmentation of TERT by caspase activation after HCV infection (Fig 6) suggested that cellular mitochondria may be involved in this process. Confocal microscopy after labelling HCV infected cells with *MitoTracker* (red) and NS3 or TERT with second antibody (green) showed that either NS3 or TERT co-labelled with *MitoTracker* (Fig 7B, middle and lower panels respectively) most intensely in a globular perinuclear pattern. In contrast, uninfected cells showed less co-labelling of TERT and *MitoTracker*, (Fig 7B, upper panel). Collectively these data indicate that HCV infection leads to increased distribution of TERT to mitochondria, similar to the TERT mitochondrial response shown to occur as a result of oxidative stress [34–36].

**Fig 8:**
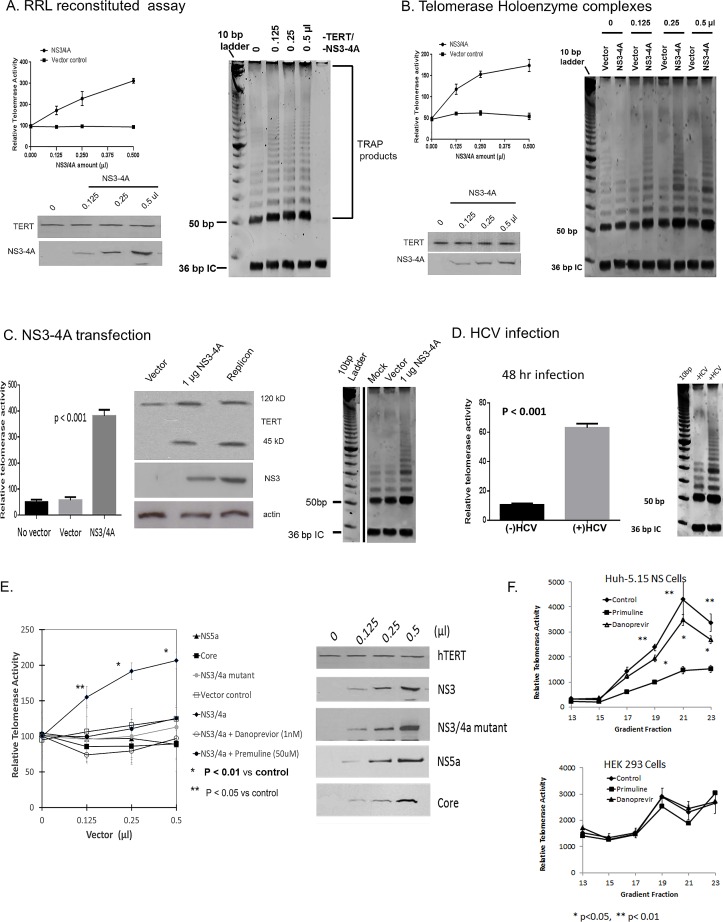
NS3-4A stimulates telomerase activity. **(A) NS3-4A stimulates activity of cell free mixtures of TERT and TERC**. NS3-4A, TERT and TERC were prepared from pcDNA 3.1 vectors using RRL cell free incubations in independent tubes. For TERC, T7 runoff transcripts were prepared from TERC DNA vector and RNA was gel purified from RRL incubations. Equivalent amounts of TERT and TERC were incubated with increasing amounts of NS3-4A or empty vector and subjected to *real-time* TRAP assay (Left panel) or TRAP products visualized on non-denaturing 12% PAGE using TRAPeze (Right Panel). WB, lower left shows TRAP loading controls. Abbreviations: bp = base pairs, IC = internal control = 36 bp. Initial product on gel is 54 bp. Note label of TRAP products. **(B) NS3-4A stimulates telomerase activity of TERT holoenzyme complexes**. Lysates from log-phase uninfected Huh-7 cells were separated on 10–30% glycerol gradients. 10 ul aliquots of gradient fractions 17–19 were mixed with various amounts of NS3-4A which were generated from cell-free RRL incubations of NS3-4A pcDNA3.1 vector. The mixtures were either assayed by *real-time* TRAP assay (Left panel) or TRAP products visualized with TRAPeze (Right panel) as described for Fig 8A. WB, lower left shows TRAP loading controls. Abbreviations: as in Fig 8A. **(C) NS3-4A transfection**. HEK-293 cells were transfected with 1ug (as DNA) of vector containing NS3-4A, empty vector, or mock control. 48 hr later, cells were lysed and telomerase activity determined with *real time* TRAP assay (Left panel) or TRAP products visualized with TRAPeze (Right panel). WB, middle panel shows TRAP loading controls. Abbreviations: as in Fig 8A. Gel lanes shown for Fig 8C were from the same gel as Fig 8B, consequently, the 10 bp ladder is identical. **(D) HCV infection.** Huh-7.5 cells were infected with HCVcc and 2 days later telomerase activity was determined with *real time* TRAP assay (Left panel) or TRAP products visualized with TRAPeze (Right panel). **(E)** Specificity of NS3-4A stimulation of telomerase. All probes were constructed as we reported previously [24]. Translational products were prepared from pcDNA 3.1 vectors using RRL cell free system in independent tubes. TERC was prepared as described in 8C, then equal amounts were added to a constant amount of TERT and various amounts of NS3-4A, core, NS5A, or NS3-4A with primuline (5 uM) (NS3 helicase inhibitor) or danoprevir (1nM) (NS3 protease inhibitor). Telomerase activity was quantified by *realtime* TRAP- RT PCR assay (upper panel). Input RRL protein products were verified by immunoblots (lower panel). [NS3-4A and TERT aliquots were from same RRL incubation as used for Fig 8A]. [NS3-4A mutant = catalytically silent protease from serine-alanine mutation (HCV H strain serine 139 to alanine)]. **(F)** Inhibition of NS3-4A-holoenzyme complexes. Holoenzyme complexes were prepared from 10–30% glycerol gradient fractions of cellular extracts obtained from HCV replicons (Huh-5-15NS Upper panel) or control HEK-293 cells. Telomerase activity was assayed in each fraction using *realtime* TRAP- RT PCR with or without 1 nm danoprevir or 5 uM primuline. [*p< 0.05 **p < 0.01 from uninhibited control reaction.]

Related SF-2 helicases, DHX 36 and DDX 39 have recently been shown to bind either the RNA component of the telomerase complex, TERC [37, 38], or the CTE region of TERT [28] respectively, and influence telomerase activity. Knowing that HCV infection could stimulate telomerase activity (Fig 2) and that NS3 could both bind to TERT and sediment with the active holoenzyme complex (Figs 4A, 4B and 5A respectively), we investigated whether NS3-4A could directly stimulate telomerase activity. We first assembled basic catalytic complexes of TERT, TERC, and NS3-4A by generation of the individual components in rabbit reticulocyte lysate (RRL) incubations. Then, aliquots were added to TRAP reaction mixtures and telomerase activity determined with *real time* PCR assay of TRAP products or visualization of products on gels (Fig 8A left and right panels respectively). These experiments demonstrated that NS3-4A added to basic complexes of TERT and TERC stimulated catalytic activity. We next added RRL generated NS3-4A complex to aliquots of uninfected Huh-7 cell holoenzyme complexes, (pooled corresponding fractions 17–19 of Fig 5D) and observed that NS3-4A also stimulated telomerase activity of the holoenzyme (Fig 8B, left and right panels). Consequently, NS3-4A is capable of stimulating the catalytic activity of either the basic telomerase complex of TERT and TERC as well as the heavy, holoenzyme; the latter containing multiple structural and regulatory components. As a comparison, NS3-4A transfection also elicited increased telomerase activity in HEK-293 cells, similar to HCV infection observed at day 3 in Huh-7.5 cells (Fig 8C and 8D respectively).

To determine the specificity of NS3-4A stimulation of the telomerase catalytic complex, RRL incubations were used to generate TERT, TERC, NS3-4A, core, NS5A, and inactive protease mutant NS3–4A (Fig 8E) [24]. Of all the proteins tested, only NS3-4A stimulated telomerase activity significantly (p < 0.01 as compared to vector only). Furthermore, the telomerase activity was inhibited by either an NS3 helicase (primuline) or protease inhibitor (danoprevir) and the silent alanine protease mutant was devoid of activity. These data suggest that NS3-4A requires helicase activity and an intact protease active site to stimulate telomerase. Finally, direct assays of telomerase activity in heavy glycerol gradient fractions from infected Huh-7.5 or uninfected control cells, conducted with or without helicase or protease inhibitors, (Fig 8F) showed findings similar to those obtained from the basic catalytic complex. However, danoprevir was clearly not as effective in the inhibition of NS3-4A in holoenzyme complexes as it was in the basic complexes (Fig 8E). We suspect there may be protease site access difficulties in the holoenzyme as compared to the basic telomerase complex.

## Discussion

Telomere repair is a vital enzyme activity for neoplastic cells and greater than 85% of cancer cells express telomerase. Even malignant cells which do not express telomerase have established alternative mechanisms for telomere repair [11]. In the human liver, patients infected with HCV showed increased hepatic TERT expression and telomerase activity [39] which was elevated even further in premalignant [40] and HCC specimens [41]. In vitro, overexpressed HCV proteins such as the nucleocapsid core and the NS3-4A protease-helicase have been shown to induce telomerase expression in permissive hepatocytes [17, 18, 42], and promote cellular immortality [43].

Our findings indicate that the telomerase system is stimulated early after HCV infection of hepatocytes. Uninfected primary human hepatocytes (PHH) did not express TERT or endogenous telomerase activity until after viral infection and then new TERT protein and TRAP activity were observed concomitant with the rise of HCV RNA (Fig 2). These data demonstrate that the virus can elicit de novo TERT expression with increased telomerase activity that is not just a function of malignant cells. We have previously reported that increased telomerase promoter activity, increased TERT mRNA and protein, and longer mean telomere lengths occur when the HCV nucleocapsid core protein is overexpressed in hepatocytes as compared to control cells [17]. Because core is a known transcriptional activator of a number of host genes [44] it is probable that the protein plays a role in the early transcriptional-related events necessary for telomerase expression that we observed to unfold after infection. In a number of systems, transcriptional activation of the TERT gene is a crucial step for cellular and viral oncogenesis [45].

In contrast to core protein, the influence of NS3-4A on telomerase activity appears to be much more direct and catalytic. We demonstrated three major criteria that support a direct effect of NS3-4A on TERT which are likely to impact telomerase activity: 1) in contrast to core and NS5A, NS3-4A specifically bound to TERT monomer and 45 kD cleavage fragment, 2) NS3-4A specifically associated with telomerase holoenzyme complexes which interact with telomeric chromosomal end structures to facilitate telomerase activity [16], 3) NS3-4A specifically increased the catalytic activity of holoenzyme as well as reconstituted complexes of TERT and TERC.

Immunoprecipitation experiments with fragments of TERT and NS3-4A showed that binding of the two proteins occurs at domain 2 of the helicase and /or protease sequences and the C-terminal RT/CTE, “thumb,” end of TERT. The binding and catalytic data clearly suggest that NS3-4A is capable of directly influencing the host telomerase system and set the stage for further evaluation with functional and kinetic studies. The 45 kD TERT fragment also bound NS3-4A in infected cell extracts (Fig 3) thus providing further support for end-labelling studies (S1 Fig) which showed that the fragment is from the C- terminal end of TERT.

The mechanism whereby NS3-4A enhances telomerase activity is potentially important for enabling the host cell to progress to HCC. Structurally, the NS3 protease active site groove is in close proximity to the “backside” of helicase domain 2 [46], and TERT thumb binding to this area would allow the crucial nucleic acid binding sites of the protease and the helicase activities of domain 2 to interact with CTE motifs of TERT that have been shown to promote RNA-DNA heteroduplex formation, active site stabilization, and increased telomerase processivity [47–49]. Our findings that the increased telomerase activity elicited by NS3-4A was inhibited with either anti-NS3 protease or anti-NS3 helicase are consistent with the binding data as well as studies demonstrating that the protease and helicase domains are interdependent [2, 3]. Helicase RNA (or DNA) binding, unwinding, and ATPase activities depend upon a functional protease groove and surrounding electropositive residues which have been proposed to optimize NTP binding interactions [50, 51]. Furthermore, the protease domain improves translocation stepping efficiency of the helicase thereby increasing processivity [52]. Because NS3-4A can unwind DNA as well as RNA, it is plausible that the helicase may interact with either the telomeric DNA substrate or the RNA template of TERT to augment telomerase activity. Changes in substrate and template orientations through alterations of nucleic acid secondary structures have been shown to markedly increase telomerase processivity [53, 54].

TERT protein is synthesized in the cytoplasm and transported to the nucleus where it is assembled in the nucleolus with TERC and other proteins into a functional high molecular weight RNP to form the proposed telomerase active holoenzyme. The holoenzyme contains additional proteins important for telomere recognition, telomerase activity, and chromosome end repair [12]. During S phase the active complex is shuttled from nucleoli to Cajal bodies to repair telomere ends [13, 55]. It is not known at this time whether NS3-4A influences assembly and structure of the holoenzyme complex; however, activity experiments did show that the protein could stimulate holoenzyme activity in a concentration dependent manor.

In cellular extracts subjected to gradient sedimentation, the majority of telomerase activity was recovered in the heavy gradient fractions with only minimal activity recovered in the lower molecular weight fractions in accordance with previous reports [15, 16]. While the heavy holoenzyme complexes exclusively contained the TERT 120 kD monomer, the lower molecular weight fractions primarily contained the more abundant 45 kD TERT cleavage fragment which is unlikely to have appreciable telomerase activity. Through end labelling studies and use of C-terminal specific anti-TERT antibodies we determined that the 45 kD fragment originates from the C-terminal end of the TERT monomer (S1 Fig). This fragment would be expected to lack the N-terminal TERT sequences that bind TERC and provide the RNA template that is required for canonical telomerase activity.

Previous studies have shown that HCV infection activates caspases presumably through NS3-4A binding to initiator Caspase 8 [31, 56]. Furthermore, TERT has been shown to be a target of effector Caspases 6, and 7 and to a much lesser extent, Caspase 3, which digest TERT monomer to fragments of similar sizes to those shown here (Fig 6) [33]. Our data are the first to show that HCV infection can activate caspases which in turn target and degrade TERT. Furthermore, of the two major effector caspases known to target TERT, only caspase *7* was activated suggesting specific activation for HCV infection. Collectively, our findings show that not only is TERT induced early in infection, but the systems responsible for TERT degradation are also activated, thus suggesting that the virus extensively influences the telomerase system. In accordance with the caspase activation studies, the immunohistochemistry data showed that HCV infection was associated with increased TERT localization in perinuclear granules, of which many co-localized with the mitochondrial marker *MitoTracker*. On the other hand, in situ localization of TERT in the nucleus was limited to scattered small foci. Although telomerase has a canonical enzymatic function that must take place in the nucleus, a growing number of reports have shown that TERT also localizes to mitochondria, especially after oxidative stress, and performs extranuclear functions [34, 35]. It is not clear at this time why our hepatoma cell lines, either infected or uninfected, contained most of the cellular TERT in the cytoplasm. Moreover, with the anti-TERT antibodies used here, it was not possible to distinguish between the 120 kD TERT monomer or the 45 kD fragment; thus, interpretation of which TERT species localizes at mitochondria is limited at this time. We are pursuing this issue with experiments employing specific end-labelling of TERT constructs. Since the 45 kD TERT fragment persists in infected cells and avidly binds NS3-4A, it is conceivable that it has extranuclear activities that may be important for oncogenesis [34].

In intact cells, canonical telomerase activity, ie, the lengthening of telomeres only occurs in the nucleus and current understanding requires TERT to be packaged in a holoenzyme complex for this to occur. The fact that NS3-4A could be recovered in holenzyme complexes of infected cells suggests that a small portion of NS3-4A enters the nucleus and influences telomerase activity. Past reports have shown a very limited potential nuclear role for NS3-4A [57] [58], however, the protease-helicase can indeed influence other nuclear proteins [59, 60]. To date, no specific host nuclear enzymatic function has been proposed for NS3-4A and our data are the first to demonstrate that the protease-helicase can impact an important nuclear enzyme system, especially one so intimately involved with the neoplasia. In our experiments, NS3-4A was not readily apparent in the nucleus, however, related SF2 helicases have broad roles in transcription, transcriptional processing, and ribonuclear protein particle assembly [61].

Only limited evidence thus far has implicated NS3-4A in oncogenic processes [62] [42]. However, closely related SF 2 RNA helicases such as the DEAD-box enzymes DDX3 and DDX5 as well as the DExD/H helicase, DHX9, have proposed roles in tumorigenesis [63, 64]. Furthermore, these enzymes have known reverse transcriptase interactions with other oncogenic viruses such as HIV and HBV. DDX3 inhibits HBV RT activity [65] and DHX9 has been shown to promote HIV RT transcription by facilitating the accessibility of viral RNA to the RT [66]. Finally, it is noteworthy that DDX3 also binds HCV core protein and facilitates cap-independent IRES–HCV RNA translation [67]. Interestingly, another related SF2 helicase, DDX39, was also shown to bind to a C-terminal region of TERT and positively regulate telomere length homeostasis [28].

In conclusion, our findings show that TERT expression and telomerase activity are stimulated early after HCV infection, and these events trigger specific caspase activation which leads to TERT degradation in a reciprocal fashion. Regulation of telomerase expression is known to be a crucial activity for neoplasia in a number of systems [12, 13]. NS3-4A appears to be a major effector for increased telomerase activity in infected cells. Immunoprecipitation and mapping experiments demonstrate that NS3-4A avidly binds to the TERT C-terminal region, associates with the telomerase holoenzyme complex, and can catalytically stimulate telomerase reverse transcriptase enzyme activity. Overall, our findings link the oncogenic enzyme telomerase with intriguing extra-viral activities of HCV which are potentially important for the neoplastic behavior of the virus. We anticipate that our findings will lead to new diagnostic, preventive, and perhaps management options for human hepatocellular carcinoma.

## Supporting Information

S1 Fig Identification of immunoreactive TERT fragments in NS 5.15 replicons and TERT overexpressed cells. Huh-7 cells were transfected with the indicated vector constructs. 48 hr later, whole cell lysates were prepared for WB analysis. Lysates were electrophoresed on parallel lanes, blotted intact, then excised and stained separately using the indicated first antibodies. Lanes were then realigned using dye front and 120 kD TERT to ensure appropriate designation of band sizes. FLAG label (1X) and HA label (1X) antigenic sites added 1130 and 1600 Daltons to the N-terminal and C-terminal ends respectively of TERT and TERT fragments. Labelled 45 kD and 50 kD fragments showed barely perceptible differences in mobility from unlabeled fragments on these gels. Abbreviations. WB Ab = western blot antibody, C = C terminal anti-TERT antibody, N = N terminal anti-TERT antibody, TERT-C-HA = Carboxy-terminal Hemagglutin label, N-FLAG-TERT = Amino-terminal FLAG label.
(TIF)

S1 Results The supporting results refer to the results depicted in S1 Fig. To further characterize lower molecular weight TERT species, we compared WB profiles of TERT using antibodies that only recognize antigenic sites on the amino (N) terminal or carboxy (C) terminal end of TERT (S1 Fig). We also prepared FLAG labelled N-terminal and hemagglutinin (HA) labelled C-terminal full length TERT vectors. Using site specific C or N terminal antibodies, WB of Huh 5.15 NS replicons showed bands at 45 kD and 50 kD respectively, and either antibody recognized 120 kD full length TERT monomer. While the 45 kD band was not seen in uninfected Huh 7.5 controls, the 50 kD band was easily identified when stained with N terminal specific antibody. Both 45 kD and 50 kD fragments were prominent in Huh 5.15 replicons and occasionally minor bands at 70–85 kD also were apparent. Cells which overexpressed TERT after full length TERT transfection also showed lower molecular weight fragments with sizes consistent with replicons. Finally, TERT overexpression by transfection of vectors containing C terminal HA or N terminal FLAG labels confirmed that the 45 and 50 kD fragments originated at the respective ends of TERT. These fragment profiles are consistent with the data of Soares et al [33] showing that TERT is a substrate for Caspases 6,7, and to a lesser extent, 3. The ability to generate both TERT fragments by overexpression-transfection virtually eliminated the possibility that the fragments were TERT alternative splicing variants, known to occur under a variety of conditions after de novo TERT transcription [68].
(DOCX)

S2 Fig Rough Uncut images for Fig 2 (TIF)

S3 Fig Rough Uuncut images for Fig 3 (TIF)

S4 Fig Rough Uuncut images for Fig 4 (TIF)

S5 Fig Rough Uncut images for Fig 5 (TIF)

S6 Fig Rough Uncut images for Fig 6 (TIF)

S7 Fig Rough Uncut images for Fig 8 (TIF)

S8 Fig Rough Uncut images for S1 Fig. (TIF)
