# Supplementary figures and images for: HCV Induces Telomerase Reverse Transcriptase, Increases Its Catalytic Activity, and Promotes Caspase Degradation in Infected Human Hepatocytes
Source: PLoS One. 2017 Jan 5;12(1):e0166853. doi: 10.1371/journal.pone.0166853 (PMC5215869; doi:10.1371/journal.pone.0166853)

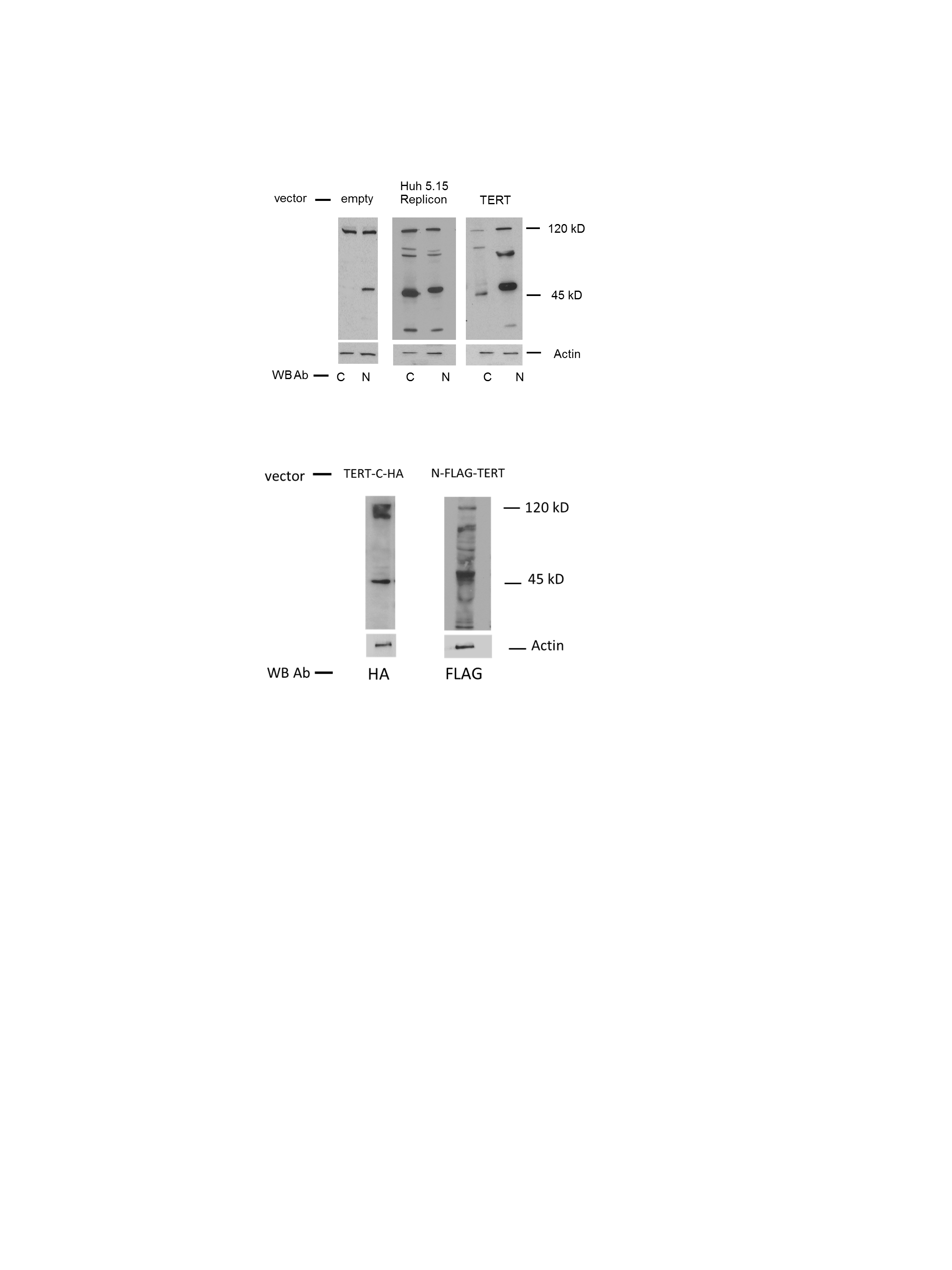

Supplement: S1 Fig — Huh-7 cells were transfected with the indicated vector constructs. 48 hr later, whole cell lysates were prepared for WB analysis. Lysates were electrophoresed on parallel lanes, blotted intact, then excised and stained separately using the indicated first antibodies. Lanes were then realigned using dye front and 120 kD TERT to ensure appropriate designation of band sizes. FLAG label (1X) and HA label (1X) antigenic sites added 1130 and 1600 Daltons to the N-terminal and C-terminal ends respectively of TERT and TERT fragments. Labelled 45 kD and 50 kD fragments showed barely perceptible differences in mobility from unlabeled fragments on these gels. Abbreviations. WB Ab = western blot antibody, C = C terminal anti-TERT antibody, N = N terminal anti-TERT antibody, TERT-C-HA = Carboxy-terminal Hemagglutin label, N-FLAG-TERT = Amino-terminal FLAG label. (TIF) [file pone.0166853.s001.TIF]

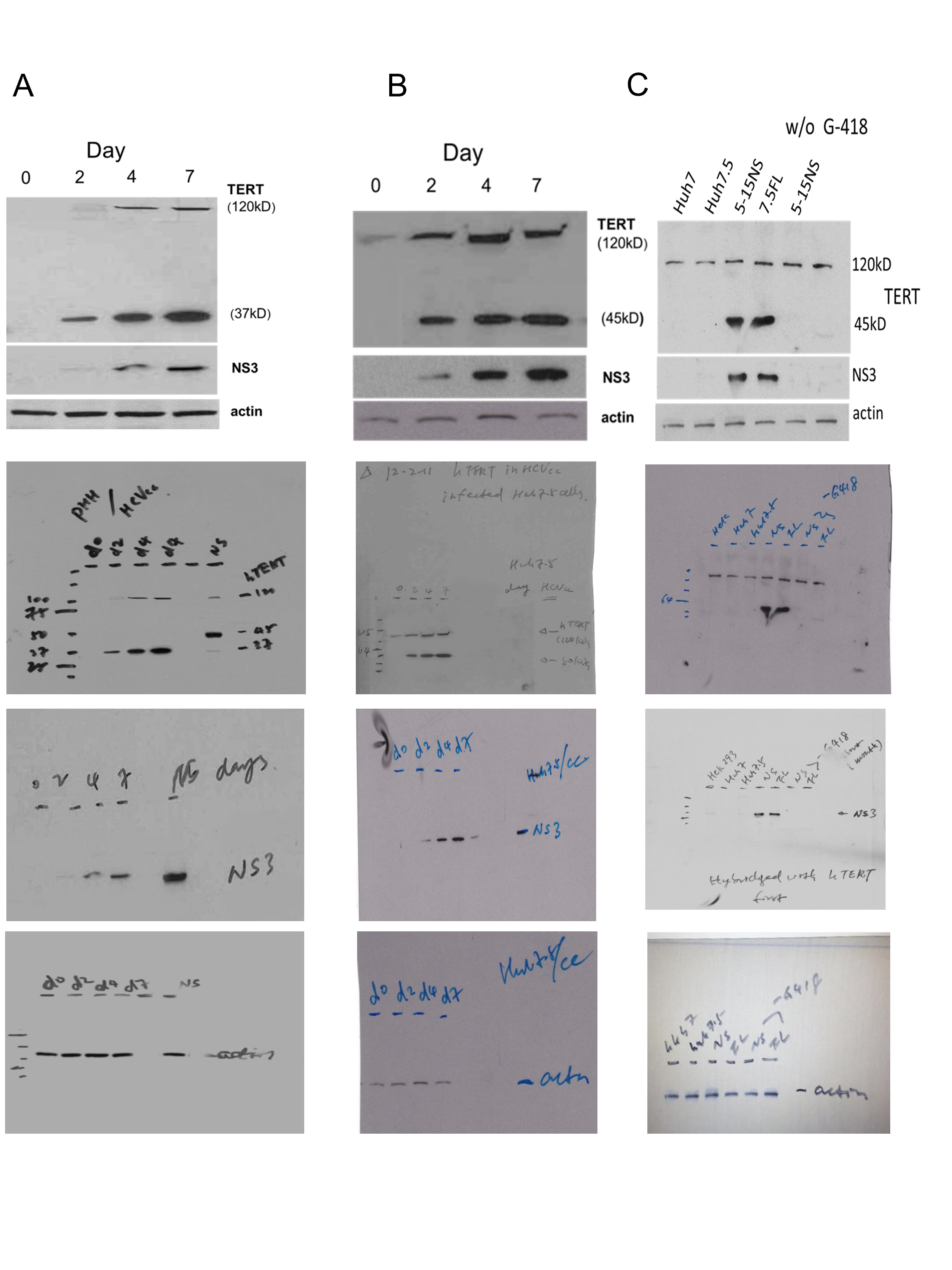

Supplement: S2 Fig — (TIF) [file pone.0166853.s003.TIF]

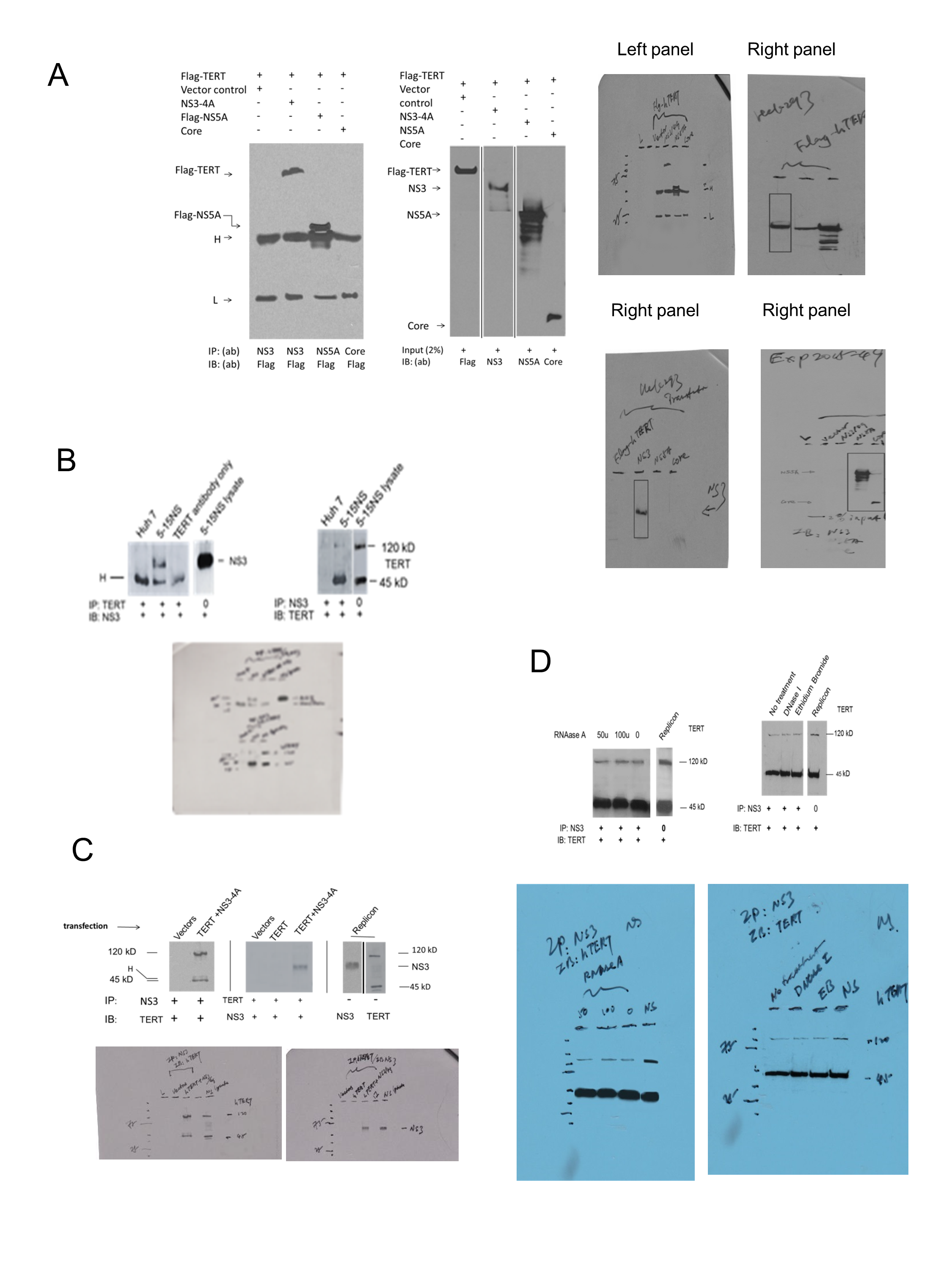

Supplement: S3 Fig — (TIF) [file pone.0166853.s004.TIF]

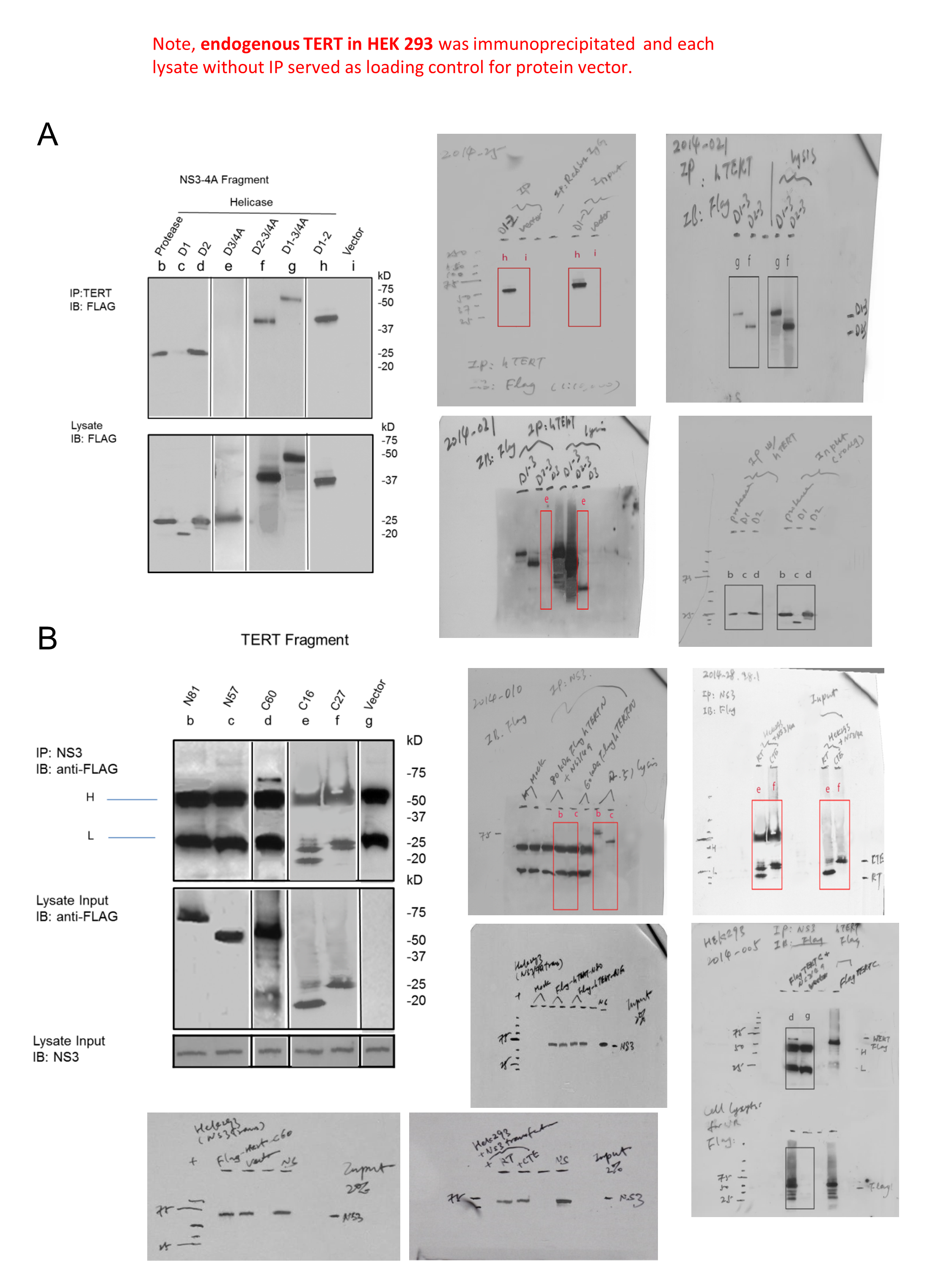

Supplement: S4 Fig — (TIF) [file pone.0166853.s005.TIF]

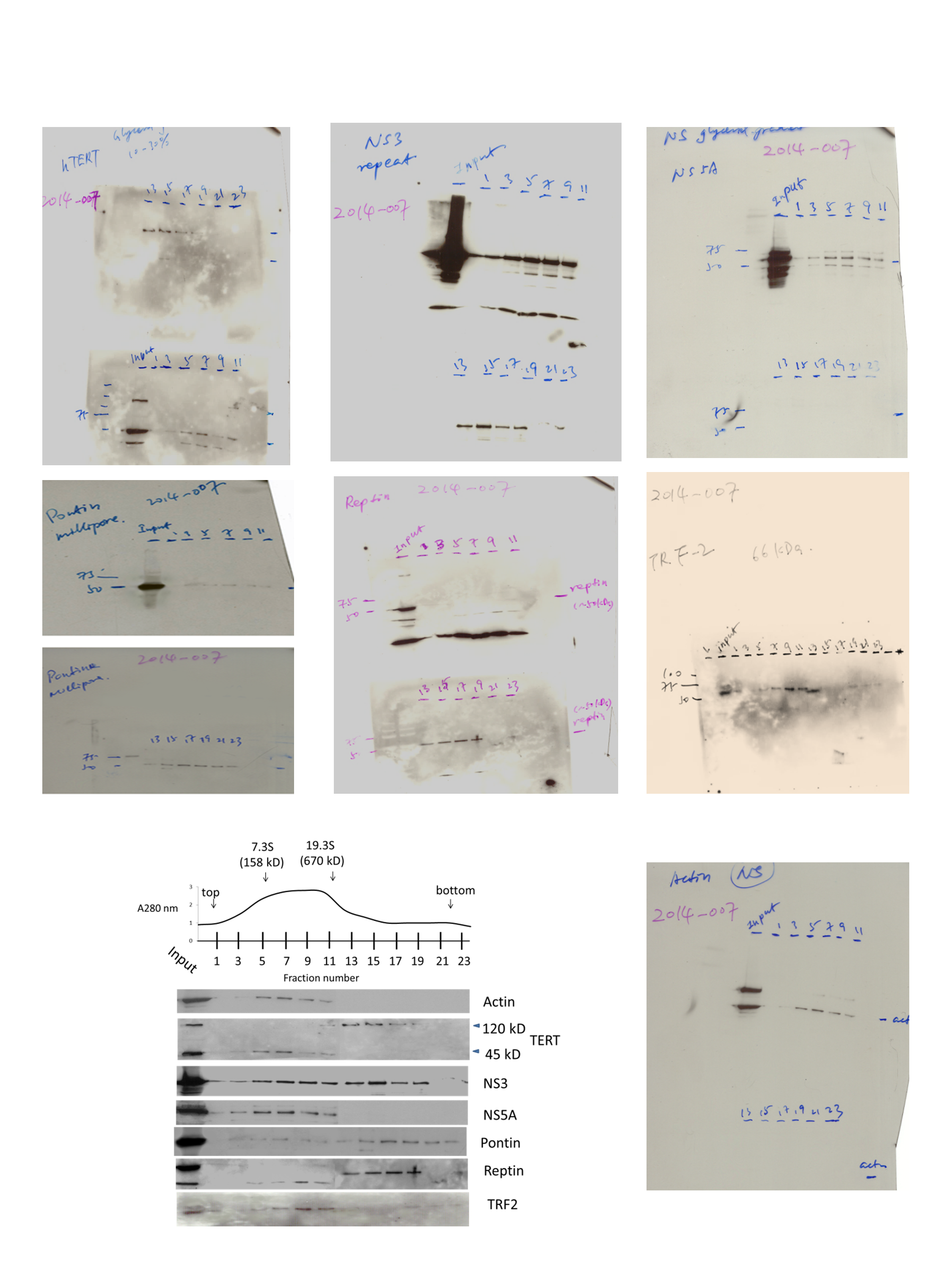

Supplement: S5 Fig — (TIF) [file pone.0166853.s006.TIF]

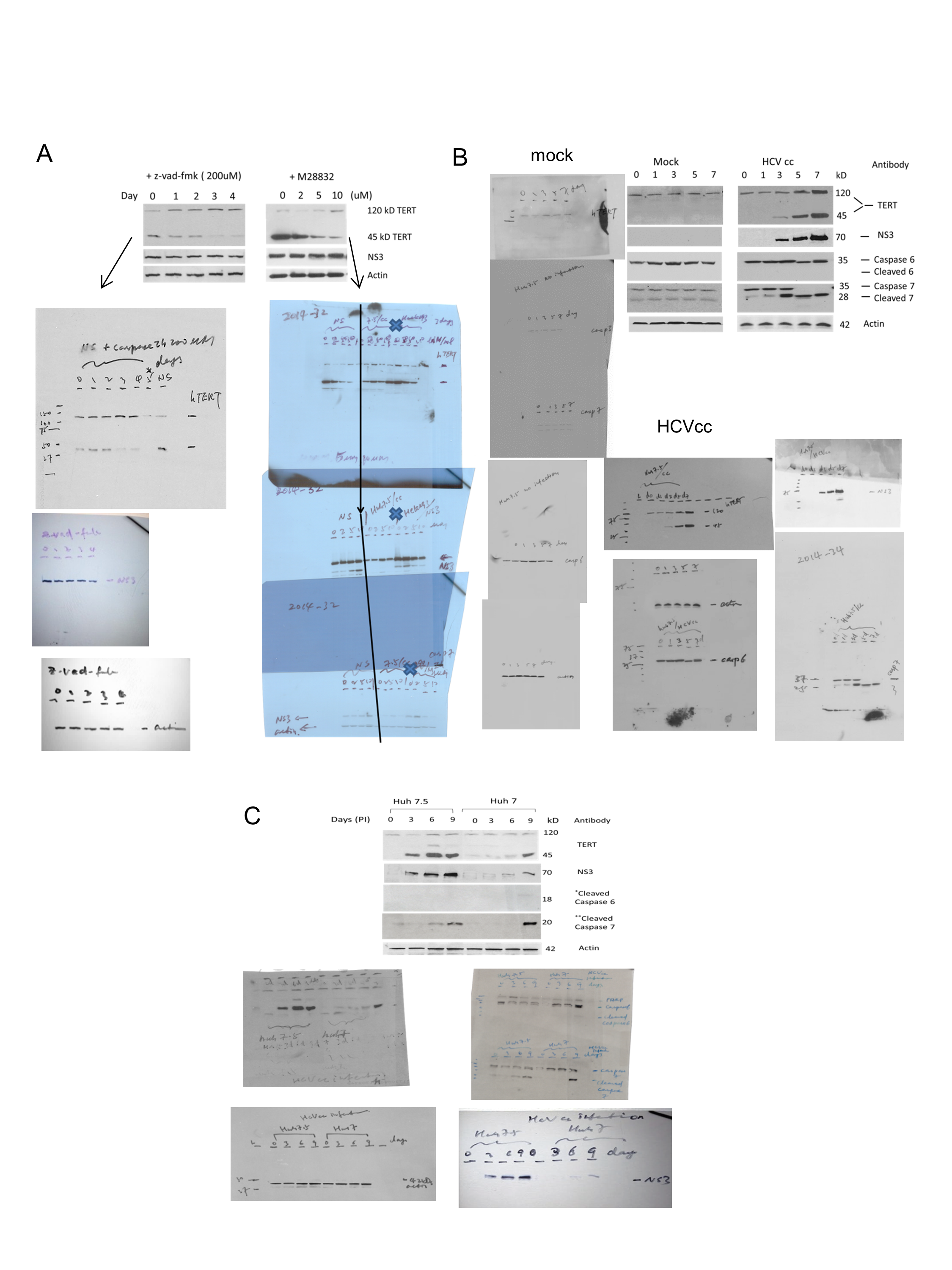

Supplement: S6 Fig — (TIF) [file pone.0166853.s007.TIF]

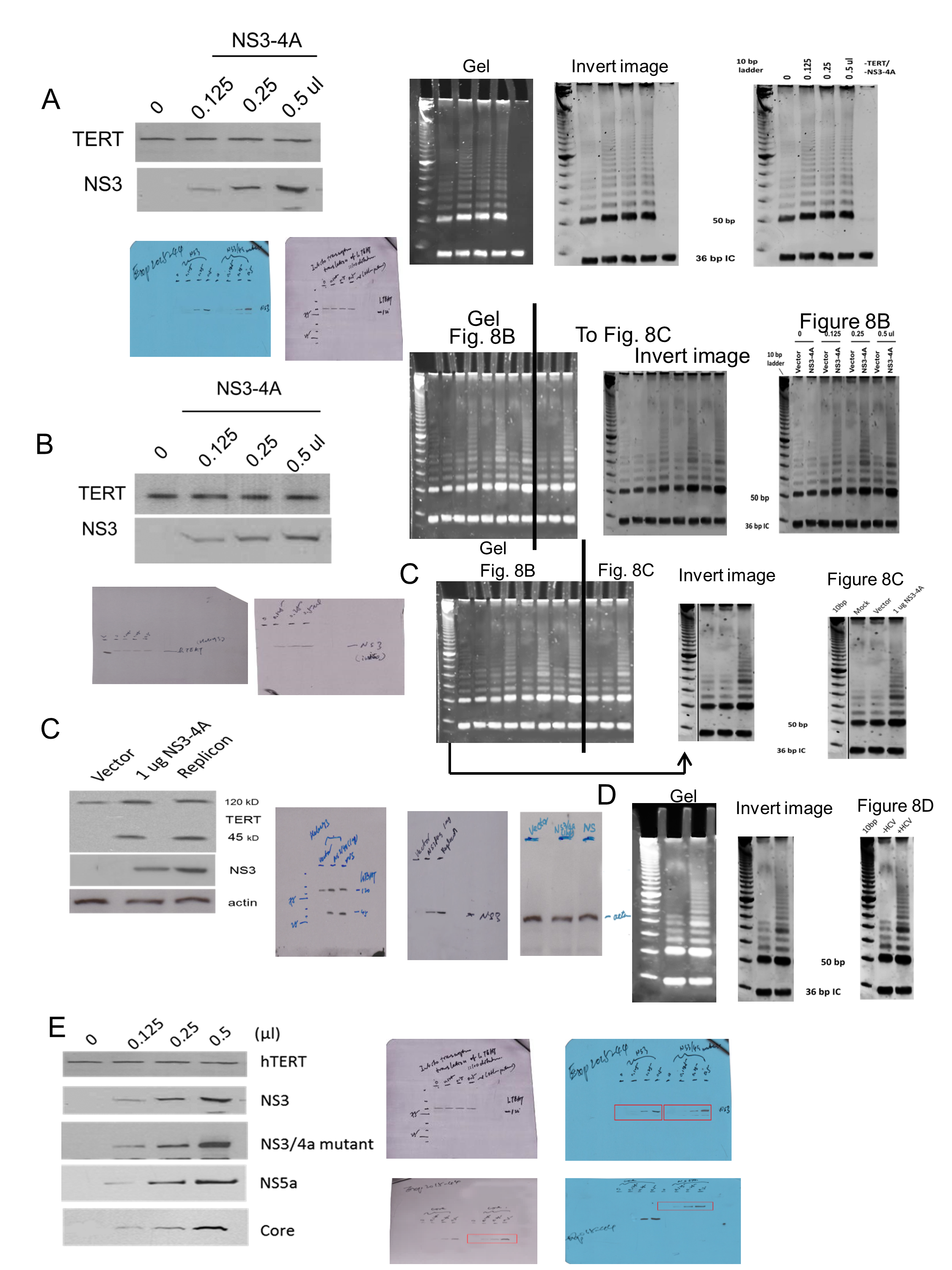

Supplement: S7 Fig — (TIF) [file pone.0166853.s008.TIF]

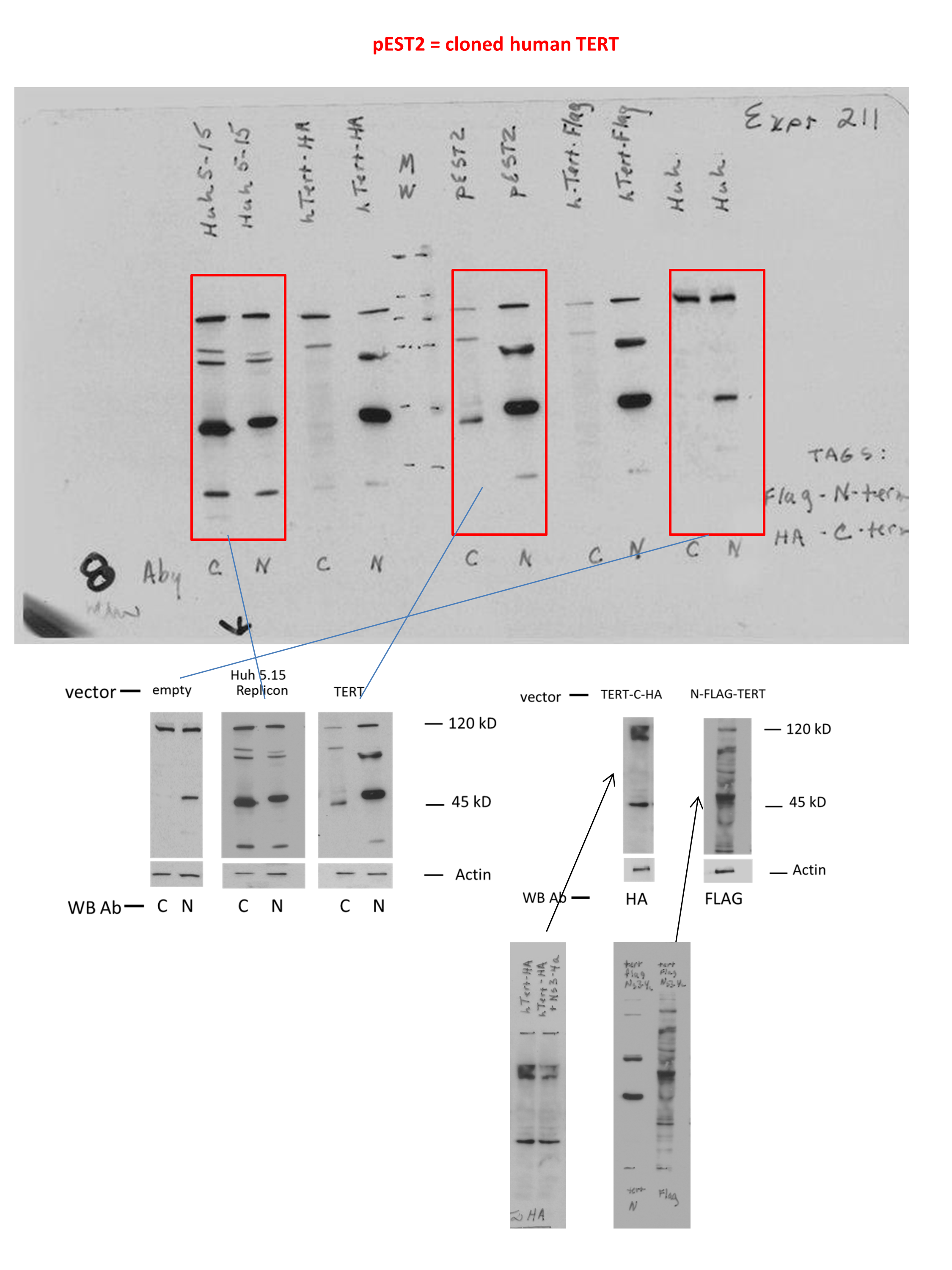

Supplement: S8 Fig — (TIF) [file pone.0166853.s009.TIF]
