# Supplementary material for: HCV Induces Telomerase Reverse Transcriptase, Increases Its Catalytic Activity, and Promotes Caspase Degradation in Infected Human Hepatocytes
Source: PLoS One. 2017 Jan 5;12(1):e0166853. doi: 10.1371/journal.pone.0166853 (PMC5215869; doi:10.1371/journal.pone.0166853)
Supplement: S1 Results — To further characterize lower molecular weight TERT species, we compared WB profiles of TERT using antibodies that only recognize antigenic sites on the amino (N) terminal or carboxy (C) terminal end of TERT (S1 Fig). We also prepared FLAG labelled N-terminal and hemagglutinin (HA) labelled C-terminal full length TERT vectors. Using site specific C or N terminal antibodies, WB of Huh 5.15 NS replicons showed bands at 45 kD and 50 kD respectively, and either antibody recognized 120 kD full length TERT monomer. While the 45 kD band was not seen in uninfected Huh 7.5 controls, the 50 kD band was easily identified when stained with N terminal specific antibody. Both 45 kD and 50 kD fragments were prominent in Huh 5.15 replicons and occasionally minor bands at 70–85 kD also were apparent. Cells which overexpressed TERT after full length TERT transfection also showed lower molecular weight fragments with sizes consistent with replicons. Finally, TERT overexpression by transfection of vectors containing C terminal HA or N terminal FLAG labels confirmed that the 45 and 50 kD fragments originated at the respective ends of TERT. These fragment profiles are consistent with the data of Soares et al [33] showing that TERT is a substrate for Caspases 6,7, and to a lesser extent, 3. The ability to generate both TERT fragments by overexpression-transfection virtually eliminated the possibility that the fragments were TERT alternative splicing variants, known to occur under a variety of conditions after de novo TERT transcription [68]. (DOCX) [file pone.0166853.s002.docx]

**Supporting Information**

**S1 Fig.** **Identification of immunoreactive TERT fragments in NS 5.15 replicons and TERT overexpressed cells**. Huh-7 cells were transfected with the indicated vector constructs. 48 hr later, whole cell lysates were prepared for WB analysis. Lysates were electrophoresed on parallel lanes, blotted intact, then excised and stained separately using the indicated first antibodies. Lanes were then realigned using dye front and 120 kDa TERT to ensure appropriate designation of band sizes. FLAG label (1X) and HA label (1X) antigenic sites added 1130 and 1600 Daltons to the N-terminal and C-terminal ends respectively of TERT and TERT fragments. Labelled 45 kDa and 50 kDa fragments showed barely perceptible differences in mobility from unlabeled fragments on these gels.

Abbreviations. WB Ab = western blot antibody, C = C terminal anti-TERT antibody, N = N terminal anti-TERT antibody, TERT-C-HA= Carboxy-terminal Hemagglutin label, N-FLAG-TERT = Amino-terminal FLAG label.

**Results S1 . The supporting results refer to the results depicted in S1 Fig.**

To further characterize lower molecular weight TERT species, we compared WB profiles of TERT using antibodies that only recognize antigenic sites on the amino (N) terminal or carboxy (C) terminal end of TERT (Fig S1). We also prepared FLAG labelled N-terminal and hemagglutinin (HA) labelled C-terminal full length TERT vectors. Using site specific C or N terminal antibodies, WB of Huh 5.15 NS replicons showed bands at 45 kDa and 50 kDa respectively, and either antibody recognized 120 kDa full length TERT monomer. While the 45 kDa band was not seen in uninfected Huh 7.5 controls, the 50 kD band was easily identified when stained with N terminal specific antibody. Both 45 kDa and 50 kDa fragments were prominent in Huh 5.15 replicons and occasionally minor bands at 70-85 kDa also were apparent. Cells which overexpressed TERT after full length TERT transfection also showed lower molecular weight fragments with sizes consistent with replicons. Finally, TERT overexpression by transfection of vectors containing C terminal HA or N terminal FLAG labels confirmed that the 45 and 50 kDa fragments originated at the respective ends of TERT. These fragment profiles are consistent with the data of Soares et al [33] showing that TERT is a substrate for Caspases 6,7, and to a lesser extent, 3. The ability to generate both TERT fragments by overexpression-transfection virtually eliminated the possibility that the fragments were TERT alternative splicing variants, known to occur under a variety of conditions after de novo TERT transcription [68].

S2 Fig. Rough

Uncut images for Fig 2

S3 Fig. Rough

Uuncut images for Fig 3

S4 Fig. Rough

Uuncut images for Fig 4

S5 Fig. Rough

Uncut images for Fig 5

S6 Fig. Rough

Uncut images for Fig 6

S7 Fig. Rough

Uncut images for Fig 8

S8 Fig. Rough

Uncut images for supplemental Fig. 1.
